# Including Total EGFR Staining in Scoring Improves EGFR Mutations Detection by Mutation-Specific Antibodies and EGFR TKIs Response Prediction

**DOI:** 10.1371/journal.pone.0023303

**Published:** 2011-08-09

**Authors:** Shang-Gin Wu, Yih-Leong Chang, Jou-Wei Lin, Chen-Tu Wu, Hsuan-Yu Chen, Meng-Feng Tsai, Yung-Chie Lee, Chong-Jen Yu, Jin-Yuan Shih

**Affiliations:** 1 Department of Internal Medicine, College of Medicine, National Taiwan University Hospital Yun-Lin Branch, Yun-Lin, Taiwan; 2 Department of Pathology, National Taiwan University Hospital, Taipei, Taiwan; 3 Institute of Statistical Science, Academia Sinica, Taipei, Taiwan; 4 Department of Surgery, National Taiwan University Hospital, Taipei, Taiwan; 5 Department of Molecular Biotechnology, Da-Yeh University, Chang-Hua, Taiwan; 6 Department of Internal Medicine, National Taiwan University Hospital, Taipei, Taiwan; University of Barcelona, Spain

## Abstract

Epidermal growth factor receptor (EGFR) is a novel target for therapy in subsets of non-small cell lung cancer, especially adenocarcinoma. Tumors with *EGFR* mutations showed good response to EGFR tyrosine kinase inhibitors (TKIs). We aimed to identify the discriminating capacity of immunohistochemical (IHC) scoring to detect L858R and E746-A750 deletion mutation in lung adenocarcinoma patients and predict EGFR TKIs response. Patients with surgically resected lung adenocarcinoma were enrolled. *EGFR* mutation status was genotyped by PCR and direct sequencing. Mutation-specific antibodies for L858R and E746-A750 deletion were used for IHC staining. Receiver operating characteristic (ROC) curves were used to determine the capacity of IHC, including intensity and/or quickscore (Q score), in differentiating L858R and E746-A750 deletion. We enrolled 143 patients during September 2000 to May 2009. Logistic-regression-model-based scoring containing both L858R Q score and total EGFR expression Q score was able to obtain a maximal area under the curve (AUC: 0.891) to differentiate the patients with L858R. Predictive model based on IHC Q score of E746-A750 deletion and IHC intensity of total EGFR expression reached an AUC of 0.969. The predictive model of L858R had a significantly higher AUC than L858R intensity only (*p* = 0.036). Of the six patients harboring complex *EGFR* mutations with classical mutation patterns, five had positive IHC staining. For EGFR TKI treated cancer recurrence patients, those with positive mutation-specific antibody IHC staining had better EGFR TKI response (*p* = 0.008) and longer progression-free survival (*p* = 0.012) than those without. In conclusion, total EGFR expression should be included in the IHC interpretation of L858R. After adjusting for total EGFR expression, the scoring method decreased the false positive rate and increased diagnostic power. According to the scoring method, the IHC method is useful to predict the clinical outcome and refine personalized therapy.

## Introduction

Epidermal growth factor receptor (EGFR), a member of the ErbB family, is a transmembrane glycoprotein [1]. Non-small cell lung cancer (NSCLC) patients with *EGFR* mutations have had a dramatic response to EGFR tyrosine kinase inhibitors (EGFR TKIs) [2], [3]. The patients who have shown a good response to EGFR TKIs have been mainly from particular groups, including female, adenocarcinoma histology, non-smokers and Asian ethnicity [3], [4], [5]. Approximately 90% of *EGFR* mutation types have been found to be a point mutation of L858R in exon 21 and an in-frame deletion in exon 19 (Del-19), especially the E746-A750 deletion [6]. They are the most well-known EGFR TKI sensitive mutations and are also known as “classical mutations”. It is important to select patients with tumors harboring *EGFR* mutations when using EGFR TKIs. For *EGFR* mutation analysis, different molecular techniques such as direct DNA sequencing and scorpion amplified refractory mutation systems (ARMS) have been used [7], but they are time-consuming, expensive and complicated, and thus not routinely used in general hospitals or clinical laboratories.

Yu et al. developed mutation-specific rabbit monoclonal antibodies against the E746-A750 deletion and L858R mutation of EGFR [8]. Immunohistochemistry (IHC) is a well-established method, and is applied broadly in routine biopsy tissue diagnosis in clinical practice. It can also be applied in small tissue samples, fine needle aspiration cytology and cell blocks from body fluids. This simple assay is a rapid and cost-effective method, and it can be used as screening to identify most candidates who may have a favorable response to EGFR TKIs [8], [9]. The sensitivity and specificity of the mutation-specific antibodies of EGFR have been confirmed [8], [10]. However, the range of the overall sensitivity has been found to be from 47% to 92% in different studies using the same antibodies [8], [11].

Although the IHC approach can support the routine assessment of specific *EGFR* mutations, different scoring schemes of IHC staining have also been adopted. Most of the published studies have used an intensity scoring method [8], [9], [10], [11], [12], although the University of Colorado's IHC H-score criteria and other scoring systems have also been adopted [13], [14]. However, no statistical methodology has been used to confirm whether or not the scoring method of IHC intensity is optimal. Furthermore, Kitamura et al. reported that a positive reaction to the two mutation-specific antibodies was associated with the expression of total EGFR by EGFR antibody [11]. However, there have not been any studies focusing on whether total EGFR expression level has any influence on the IHC interpretation of the two EGFR mutation-specific antibodies.

The prior reports have shown variable sensitivity and specificity to detect activating *EGFR* mutations by the EGFR mutation-specific antibodies[8], [10], [11], [13], [14]. In addition, the role of IHC-based *EGFR* mutations to predict clinical response and progression free survival to EGFR TKIs was still controversial [11], [13], [14]. For this reason. the aim of this study was to identify the discriminating capacity of IHC scoring for the detection of the two specific *EGFR* mutations, L858R and E746-A750 deletion, in patients with adenocarcinoma of the lung. The impact of total EGFR expression was considered into the analysis of the scoring assessment. The clinical outcomes, including time to tumor recurrence and EGFR TKI treatment outcomes were also studied.

## Materials and Methods

### Patients and tissue procurement

We collected surgically resected lung tumors at the National Taiwan University Hospital (Taipei, Taiwan) from September 2000 to May 2009. Patients with paraffin-embedded surgically resected lung tumor specimens, histologically confirmed lung adenocarcinoma were included. Informed consent about the use of these specimens for future molecular studies was obtained before surgery after approval of the Institutional Review Board (IRB). (the IRB approval number: 993703374) The paraffin-embedded tissues were collected for *EGFR* sequencing and IHC staining of EGFR mutation-specific antibodies.

The histology of lung cancer was classified according to the World Health Organization pathology classification [15]. All of the lung cancer patients received complete lung cancer staging work-up as a routine practice before surgery, which included computed tomography (CT) of the head, chest and abdomen, and whole body bone scintigraphy. The disease stage was determined by the Tumor-Node-Metastasis system for NSCLC staging [16]. The dates of diagnosis, surgical excision, tumor recurrence and survival were recorded. All systemic treatments as adjuvant treatment or after tumor recurrence, including chemotherapy and EGFR TKIs, and responsiveness to the treatment were recorded.

Clinical data, including demographic information and smoking status, were recorded, and imaging studies were collected. Smoking status was defined as non-smokers (<100 cigarettes in the patient's lifetime), current smokers (patients smoking with 1 year of diagnosis), and former smokers (all others).

### Response evaluation of lung adenocarcinoma patients

We reviewed all patients' image studies during the whole disease course. The unidimensional method was used according to the Response Evaluation Criteria in Solid Tumor guidelines to evaluate measurable solid tumors [17]. Only patients with a complete response (CR) and partial response (PR) were regarded as responders. Time to tumor recurrence was measured from the date of operation until the first date of tumor recurrence via imaging studies. Progression-free survival (PFS) was calculated from the date of initiation of the EGFR TKI treatment until the first objective or clinical sign of disease progression or death.

### Sequencing of EGFR exons 18–21

The surgically resected tumor sections were first evaluated with hematoxylin and eosin staining. Macrodissection was performed to make the tissue samples consist of more than 80% cancer cells. DNA was extracted using a QIAmp DNA Mini Kit (Qiagen, Valencia, CA) for EGFR mutation analysis. The tyrosine kinase domain of the EGFR coding sequence, exons 18, 19, 20, and 21 were amplified by nested PCRs from DNA, and PCR amplicons were purified as described previously [18], [19], [20].

PCR amplicons were sequenced in both the sense and antisense directions and chromatograms were examined manually. *EGFR* mutations detected in the initial round of sequencing were confirmed by independent PCR and sequencing reactions. Only specimens in which a mutation was identified in both rounds were recorded as mutation-positive.

Complex *EGFR* mutations were defined as two or more concomitant different *EGFR* mutations. When the complex *EGFR* mutation had either an in-frame Del-19 or a point mutation L858R in exon 21, it was defined as a “classical mutation pattern” [21].

### Immunohistochemistry for EGFR mutations

4-µm sections were cut from the paraffin-embedded tissue samples of the surgically resected lung adenocarcinomas. Antigen retrieval by AR-10 Solution (EDTA buffer) (Biogenex San Ramon, CA) was performed at 121°C for 10 minutes. The commercial monoclonal antibodies, including L858R (clone 43B2) and E746-A750 deletion (clone 6B6) (Cell Signaling Technology, Danvers, MA), were applied (dilution 1∶150 for both antibodies) and the slides incubated overnight at 4°C. A diaminobenzidine (DAB) (BioGenex, San Ramon, CA) detection kit was used with extra washing steps selected. The slides were then counter-stained with hematoxylin for 3 minutes. We also performed IHC staining for total EGFR protein using the monoclonal EGFR mouse antibody (clone 31G7, dilution 1∶150, Invitrogen, CA). Control IHC staining for pan-cytokeratin was performed using the anti-cytokeratin cocktail (clones AE1/AE3, dilution 1∶100, BioGenex, CA) according to the manufacturer's instructions.

### IHC scoring assessments

The intensity and percentage of IHC staining were recorded. The intensity was scored from 0 to 3+ and defined as follows: 0, no staining; 1+, weak staining; 2+, moderate staining; 3+, strong staining based on the staining score [8].

In addition, the quickscore (Q score) based on estimating the percentage (P) of tumor cells showing characteristic staining (0–100%) and by estimating the intensity (I) of staining was adopted for IHC scoring. The slides were scored by multiplying the percentage of positive cells by the intensity (Q = P×I; maximum  = 300) [22].

An overview of the IHC for all tissue sections was performed by two pathologists (YL Chang and CT Wu). Two observers evaluated the staining results independently and differences in interpretation were resolved by consensus.

### Statistical analysis

All categorical variables were analyzed with Pearson's χ2 tests, except where a small size required the use of Fisher's exact test. Univariate analysis of the patient characteristics was used to identify the predictive factors of EGFR TKI response. The time to tumor recurrence, PFS curve were plotted by the Kaplan-Meier method and compared by a log-rank test. Multivariate analysis for PFS was performed using Cox linear regression method. Two-sided p values of less than 0.05 were considered significant. A binary logistic regression model based on the IHC intensity/Q score of *EGFR* mutation-specific antibodies and total EGFR expression was used to predict the probability of L858R/E746-A750 deletion from direct sequencing. Receiver operating characteristic (ROC) curves were constructed for the different scoring methods. The best area under the ROC curves (AUC) was used to determine the best IHC scoring system and the correlative optimal cut-off point of IHC staining. All analyses were performed using the SPSS software package (version 17.0 for Windows; SPSS Inc.) and SAS 9.2 (SAS Institute Inc.).

## Results

### Clinical characteristics and EGFR DNA sequencing status of the lung adenocarcinoma patients

A total of 157 paraffin-embedded specimens of surgically resected lung adenocarcinomas were consecutively collected from September 2000 to May 2009. No patients received neoadjuvant chemotherapy before the tumor resection. There were 23 patients who received adjuvant chemotherapy. Fourteen sections had less than 60% tumor cells and were therefore excluded. In total, 143 samples were enrolled in this study.

Of the 143 adenocarcinoma patients, there were 72 females and 71 males with a median age of 65.2 years (range: 27.2–86.9 years). Ninety-four patients (65.7%) were non-smokers. The clinical characteristics of these patients are shown in Table 1. DNA sequencing showed *EGFR* mutations in 93 patients (65.0%), including 41 Del-19 (28.7%), 43 L858R (30.1%) and 8 other mutations (5.6%). Thirty-one of the 41 Del-19 patients (75.6%) had a deletion in the range of E746-A750 (Supporting Information Table S1). Females (female: 79.2% versus (vs.) male: 50.7%; *p<*0.001) and non-smokers (non-smokers: 77.7% vs. former/current smokers: 40.8%; *p<*0.001) had higher *EGFR* mutation rates.

**Table 1:** Clinical characteristics and *EGFR* DNA sequencing results.

Variable		Total patients	(%)
**Total No.**		143	100
**Age median**		65.2	
**(range)**		(27.2–86.9)	
**Sex**			
	**Female**	72	50.3
	**Male**	71	49.7
**Smoking**			
	**Non-smoker**	94	65.7
	**Former/current smoker**	49	34.3
**Initial Stage**			
	**I**	74	51.7
	**II**	21	14.7
	**III**	46	32.2
	**IV^<$>\raster="rg1"<$>^**	2	1.4
***EGFR*** ** mutation**			
	**E746-A750 deletion**	31	21.7
	**Other Del-19**	10	7.0
	**L858R** *	43	30.1
	**Others#**	8	5.6
	**Wild**	50	35.0

*Including: two L858R+V834L, one L858R+E709V, one L858R+T790M and one L858R+ K757N.

#Including: two L861Q, one E709K+G719A, one E709K+G719S, one G719A+L861Q, one N771-H773 dupNPH, one K860I+861Q, and one R831C+L861R.

<$>\raster="rg1"<$>Two patients received cranial tumor excision for solitary brain metastasis and lobectomy for a pulmonary nodule.

### IHC results of lung adenocarcinomas

**Figure 1:**
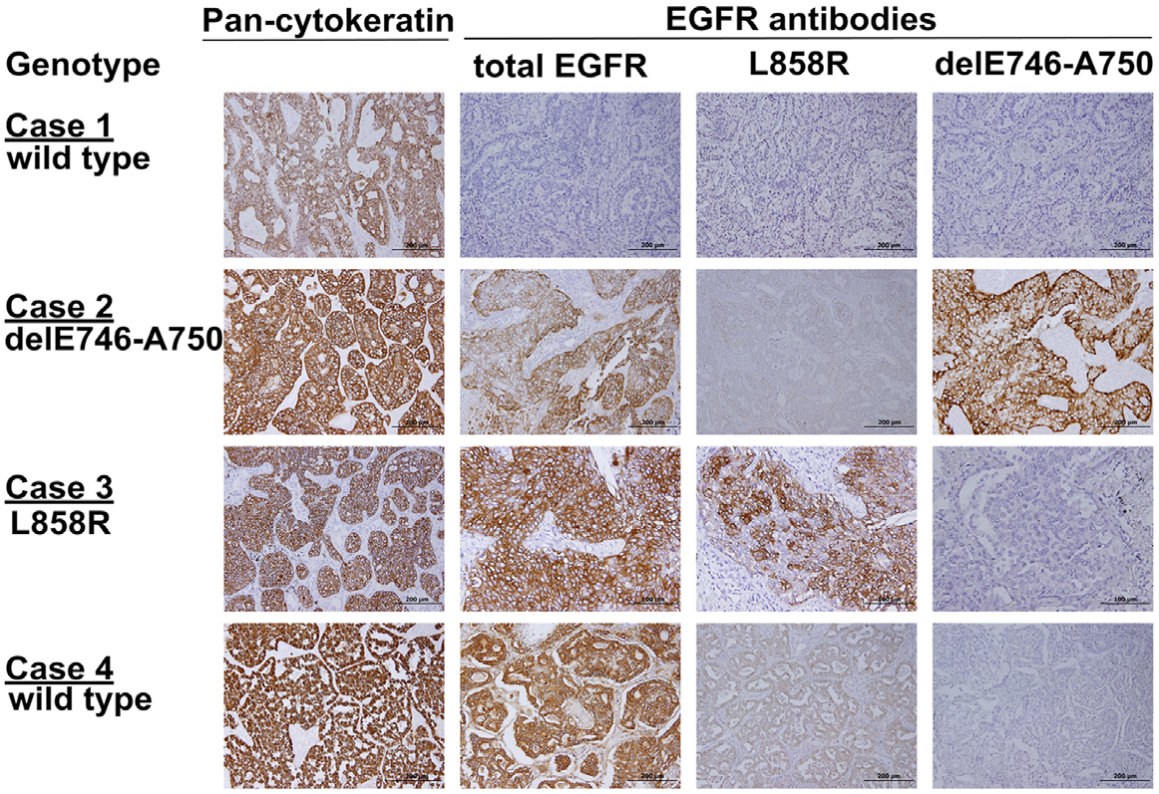
Immunohistochemical stain of lung adenocarcinoma. Control pan-cytokeratin antibody stains all tissue samples regardless of EGFR mutation status. Case 1. A sample with wild-type *EGFR* was not stained with total EGFR, L858R and delE746-A750 antibodies. Case 2. A sample with delE746-A750 was stained with both total EGFR and delE746-A750 specific antibody. Case 3. A sample with L858R was stained with both total EGFR and L858R specific antibody. Case 4. A sample with wild-type *EGFR* was stained with moderate intensity of total EGFR and mild intensity of L858R specific antibody.

The mutation-specific antibodies (L858R point mutation and E746-A750 deletion in exon 19), total EGFR antibody and pan-cytokeratin were used to stain the 143 tumor sections. IHC with pan-cytokeratin was positive to confirm the reactivity of the tissues for IHC and verify the quality of theses tissue samples (Figure 1). The mutation-specific antibodies and total EGFR antibody have distinct immunoreactivity for the tumor cells as presented in Figure 1. The IHC intensity, Q score and the predictive probability of the logistic regression model based on specific antibodies and total EGFR expression were used to construct the ROC curves and calculate the AUCs (Table 2).

**Table 2:** The diagnostic power of immunohistochemical scoring methods for predicting L858R and E746-A750 deletion by *EGFR* mutation-specific antibodies.

Item	ROC area (95% CI)	Optimal cut-off point						
		Sensitivity	Specificity	PPV	NPV	LR+	LR-	Diagnosticaccuracy
**L858R intensity**	0.853(0.785–0.922)	76.7%	82.0%	64.7%	89.1%	4.261	0.284	80.4%
**L858R Q score**	0.867(0.801–0.933)	81.4%	78.0%	61.4%	90.7%	3.700	0.238	79.0%
**L858R intensity + total EGFR intensity** *	0.859(0.788–0.931)	79.1%	80.0%	63.0%	89.9%	3.955	0.261	79.7%
**L858R intensity + total EGFR Q score** *	0.876(0.811–0.941)	86.0%	78.0%	62.7%	92.9%	3.909	0.179	80.4%
**L858R Q score + total EGFR intensity** *	0.862(0.790–0.934)	88.4%	76.0%	61.3%	93.8%	3.683	0.153	79.7%
**L858R Q score + total EGFR Q score** *	0.891(0.830–0.953)	88.4%	77.0%	62.3%	93.9%	3.843	0.151	80.4%
**delE746-A750 intensity**	0.958(0.000–1.000)	93.5%	94.6%	82.9%	98.1%	17.315	0.069	94.4%
**delE746-A750 Q score**	0.960(0.000–1.000)	93.5%	94.6%	82.9%	98.1%	17.315	0.069	94.4%
**delE746-A750 intensity + total EGFR intensity** *	0.950(0.871–1.000)	93.5%	95.5%	85.3%	98.2%	20.778	0.068	95.1%
**delE746-A750 intensity + total EGFR Q score** *	0.958(0.897–1.000)	93.5%	95.5%	85.3%	98.2%	20.778	0.068	95.1%
**delE746-A750 Q score + total EGFR intensity** *	0.969(0.915–1.000)	93.5%	94.6%	82.9%	98.1%	17.315	0.069	94.4%
**delE746-A750 Q score + total EGFR Q score** *	0.959(0.890–1.000)	93.5%	95.5%	85.3%	98.2%	20.778	0.068	95.1%

ROC = receiver operating characteristic, CI =  confidence interval, PPV =  positive predictive value, NPV =  negative predictive value, LR+ =  positive likelihood ratio, LR- =  negative likelihood ratio.

The optimal cut-off point was defined as the one with the least (1 - sensitivity)^2^ + (1 - specificity)^2^.

*Logistic regression model to evaluate the predicted probability of the interaction between the two IHC scoring system.

For IHC staining of L858R, the best AUC came from the predictive probability of the logistic regression model based on the L858R Q scores and the total EGFR expression Q scores (Table 2). The best AUC of L858R was 0.891, and the correlative optimal cut-off point was 0.154 of the predictive probability by the logistic regression model. According to the optimal cut-off point, the L858R IHC staining scoring method, which combined L858R Q scores with total EGFR expression Q scores, showed 88.4% sensitivity, 77.0% specificity, 62.3% positive predictive value (PPV), and 93.9% negative predictive value (NPV).

For the E746-A750 deletion, the best AUC came from the predictive probability of the logistic regression model based on the IHC Q score of E746-A750 deletion and the IHC intensity of total EGFR expression (Table 2). The best AUC was 0.969, and the correlative optimal cut-off point was 0.061 of the predictive probability by the logistic regression model. According to the optimal cut-off point, the E746-A750 deletion scoring method, which combined Q scores of E746-A750 and the intensity of total EGFR expression, showed 93.5% sensitivity, 94.6% specificity, 82.9% PPV, and 98.1% NPV.

IHC staining with the L858R mutation-specific antibody was detected in 38 of 43 L858R-mutated cases who were proven by DNA sequencing. Of the 31 patients with the E746-A750 deletion by DNA sequencing, IHC staining with the E746-A750 deletion mutation-specific antibody was detected in 29 patients. For the 10 patients with deletions in exon 19 other than the E746-A750 deletion, one patient with the L747-T751 deletion in exon 19 was also positive for IHC staining by the E746-A750 deletion specific antibody (Table 3 and Table S1).

**Table 3:** Comparison of results of *EGFR* mutation-specific antibodies and DNA direct sequencing.

IHC for L858R and E746-A750 (N = 143)			
IHC L858R	DNA sequencing for L858R		
	L858R	Non-L858R	Total
**Positive**	38	23	61
**Negative**	5	77	82
**Total**	43	100	143

IHC delE746-A750	DNA sequencing for Exon 19			
	delE746-A750	other	Non-del 19	Total
**Positive**	29	1	5	35
**Negative**	2	9	97	108
**Total**	31	10	102	143

If all deletions in exon-19 in addition to E746-A750 were considered, the sensitivity, specificity, PPV, and NPV were 73.2%, 95.1%, 85.7%, and 89.8%, respectively (Table 3 and 4). In order to detect the common *EGFR* mutations for clinical practice, the sensitivity and specificity of double staining, including E746-A750 or L858R, were 90.5% and 73.9%, respectively. 83.3% sensitivity and 74.6% specificity for the detection of any deletion in exon-19 or L858R were noted (Table 4).

**Table 4:** The detection accuracy for the *EGFR* mutation-specific antibodies.

Genotype	Sensitivity (%)	Specificity (%)	PPV(%)	NPV(%)
**L858R**	88.4%	77.0%	62.3%	93.9%
**delE746-A750**	93.5%	94.6%	82.9%	98.1%
**All Del-19**	73.2%	95.1%	85.7%	89.8%
**delE746-A750 or L858R**	90.5%	73.9%	78.8%	87.9%
**del-19 or L858R**	83.3%	74.6%	82.4%	75.9%

PPV: positive predictive value; NPV: negative predictive value.

There were 11 cases with complex mutations, and six patients with complex mutations with classical mutation patterns. The *EGFR* mutations of these six cases were L858R combined with another mutation type (Table S1). Five of the six cases had positive IHC staining with the L858R mutation-specific antibody.

### Comparison between the IHC intensity and the scoring system with best AUC

**Figure 2:**
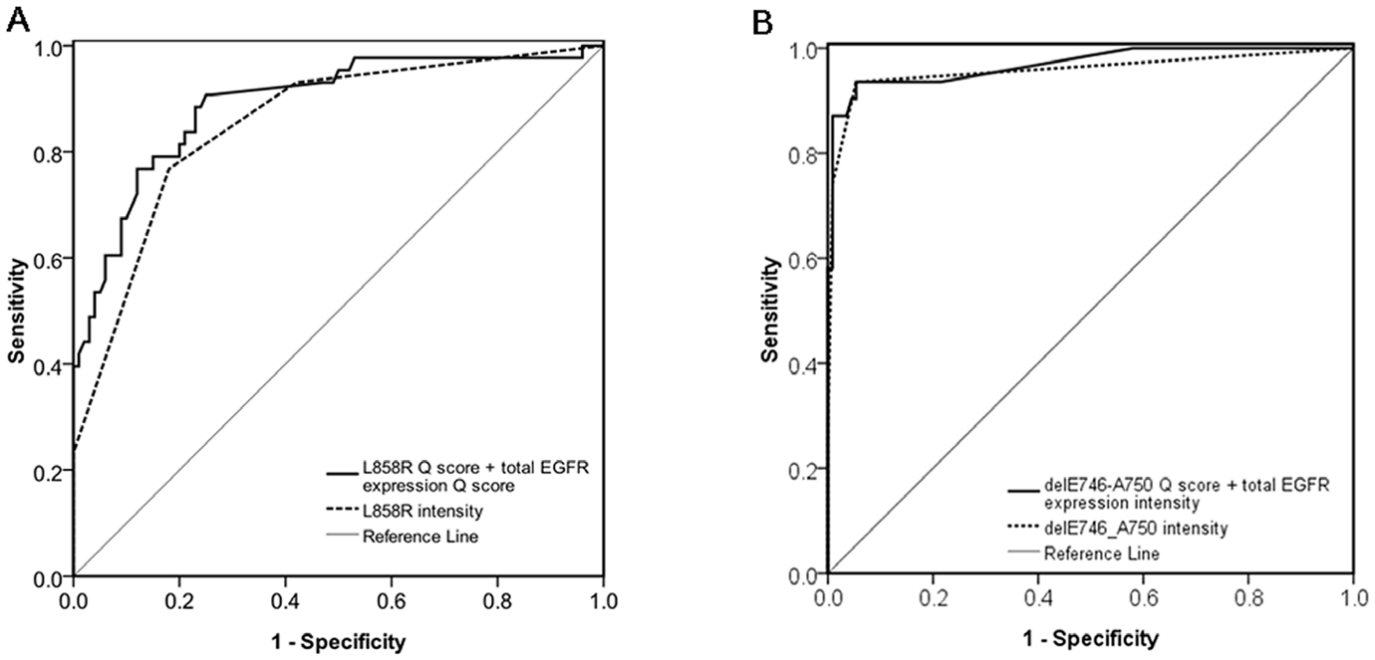
Receiver–operator characteristic (ROC) curve of *EGFR* mutation-specific antibodies IHC in predicting L858R or E746-A750. (A) AUC for the logistic regression model based on L858R Q score and total EGFR expression Q score was higher than that for L858R intensity only (0.891 vs. 0.853; *p* = 0.036). (B) the logistic regression model based on delE746-A750 Q score and total EGFR expression intensity had a trend of higher AUC than that for delE746-A750 intensity only (0.969 vs. 0.958; *p* = 0.087). AUC: area under the ROC curve.

For L858R, the best AUC came from the logistic regression model based on the L858R Q scores and the total EGFR expression Q score, and was higher than that of L858R IHC intensity only (0.891 vs. 0.853; *p* = 0.036) (Figure 2A). For E746-A750 deletion, the AUC difference did not reach statistical significance, although the logistic regression model based on E746-A750 Q score and total EGFR expression IHC intensity had a higher AUC than E746-A750 intensity only. (0.969 vs. 0.958; *p* = 0.087) (Figure 2B).

### Corresponding table for EGFR mutation status

According to the scoring method with the best AUC and the correlated best cut-off point, Table S2 and Table S3 of the Supporting Information illustrate the *EGFR* genotype based on the probability of L858R and E746-A750 deletion according to the logistic regression model with best AUC. The detailed predictive probabilities for L858R and E746-A750 are listed in Table S4 and Table S5 (Supporting Information).

### Clinical outcome of the lung adenocarcinoma patients

Of the 143 patients, 80 patients suffered from tumor recurrence. The median time to tumor recurrence was 33.4±5.8 (median ± standard error (SE)) months. According to univariate analysis, tumor size (T1, T2, T3 or T4) (*p* = 0.032), lymph node involvement (N0, N1 and N2) (*p*<0.001) and initial stage (stage I, II, III or IV) (*p* = 0.001) were the factors which had significant impacts for tumor relapse. In addition, the patients with adjuvant chemotherapy had less median time to tumor relapse than the patients without adjuvant chemotherapy (19.0 vs.41.9 months, *p* = 0.014).

However, there was no difference in time to tumor recurrence between the patients with IHC positive tumor and those with IHC negative tumor (IHC (+): 34.5 months vs. IHC (-): 33.4 months; *p* = 0.742). Sex, age(< = 65 or >65 years), smoking history and EGFR mutation status were also not the factors that significantly affected time to tumor relapse for the 143 lung adenocarcinoma patients by univariate analysis.

### Clinical treatment outcomes of EGFR TKIs in the lung adenocarcinoma patients

Among the 80 patients with tumor recurrence, 37 patients received EGFR TKIs as the systemic treatment. Twenty-five patients took gefitinib (250 mg/day) and 12 patients took erlotinib (150 mg/day). EGFR TKIs were used as first-line treatment for 7 patients (18.9%), second-line treatment for 12 patients (32.4%), third-line treatment for 14 patients (37.8%), and subsequent-line treatment for 4 patients (10.8%). No concurrent chemotherapy or radiotherapy for the lung tumors was performed during EGFR TKI therapy. Twenty-two patients (59.5%) had a partial response as maximal response, 4 patients (10.8%) had stable disease and 11 patients (29.7%) had progressive disease. The median follow-up duration for the PFS analyses was 28.3±8.9 (median ± SE) months.

The *EGFR* mutations consisted of 14 wild type, 12 Del-19 (including 10 E746-A750 deletions, one L747-P753 deletion and one delE746-T751 insQ), nine L858R, one delE709-T710 insD and one R831C + L861R (Table S6). According to the DNA sequence results, the *EGFR* mutation-positive patients (n = 23) had a longer PFS than the mutation-negative patients (n = 14) (median, 12.0 months vs. 1.7 months; *p*<0.001).

**Figure 3:**
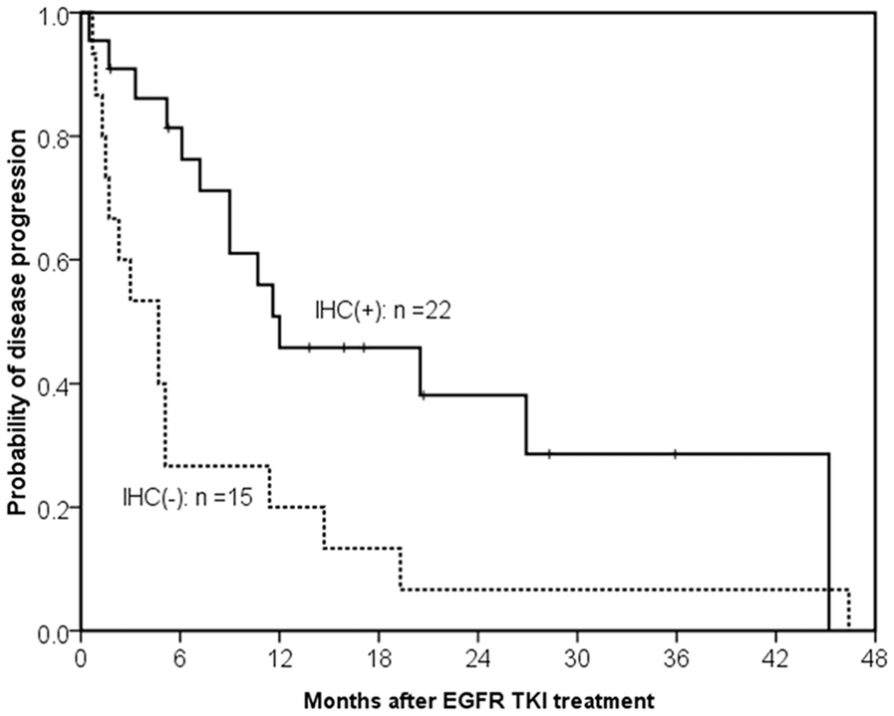
Kaplan-Meier survival curve of progression-free survival after EGFR TKIs. The patients with tumors with positive stains of *EGFR* mutation (solid line, N = 22) had a longer progression-free survival than those with negative stains of *EGFR* mutation (dashed, N = 17) (median, 12.0 months vs. 4.7 months; *p* = 0.012, by the log-rank test).

According to our scoring method with the best AUC, 22 patients had tumors with positive IHC staining and 15 patients had tumors with negative IHC staining. Of the 22 cases scored positive with IHC staining, 12 cases were scored positive with the L858R antibody, 9 cases with the E746-750 deletion antibody, and one case harboring the E746-A750 deletion by direct sequence was scored positive with both the L858R and E746-A750 deletion antibodies. The patients in the positive IHC staining group had a better response rate than those in the negative IHC staining group (77.3% (17 of 22) vs. 33.3% (5 of 15); *p* = 0.008). In addition, the patients in the positive IHC group had a longer PFS than those in the negative IHC group (median, 12.0 months vs. 4.7 months; *p* = 0.012, by the log-rank test) (Figure 3).

Multivariate analysis was performed by the Cox regression model for the potential factors, including sex, smoking, ECOG PS and EGFR IHC results (positive or negative) and line of EGFR TKI treatment. As a result, ECOG PS 2–4 (Hazard ratio (HR): 5.52, 95% confidence interval (CI): 2.04–14.95; *p* = 0.001) and positive staining of EGFR IHC (HR: 0.29, 95% CI: 0.12–0.68; *p* = 0.004) were the independent factors that significantly affected PFS for the lung adenocarcinoma patients treated with EGFR TKIs (Table 5).

**Table 5:** Multivariate analysis of progression-free survival of the 37 adenocarcinoma patients treated with EGFR TKIs.

Factors	PatientNumber	Univariate analysis	Multivariate analysis	
		*p* value	HR(95% CI)	*p* value
**Gender**				
**Female**	19			
**Male**	18	0.841	1.04(0.22–4.88)	0.963
**Smoking**				
**Never smokers**	23			
**Current/Former**	14	0.776	0.88(0.17–4.46)	0.873
**ECOG PS**				
**0**–**1**	28			
**2**–**4**	9	0.001	5.52(2.04–14.95)	0.001
***EGFR*** ** IHC** *				
**Negative**	15			
**Positive**	22	0.012	0.29(0.12–0.68)	0.004
**EGFR TKI**				
**1^st^-line**	7			
≥**2^nd^-line**	30	0.939	2.27(0.80–6.48)	0.126

*EGFR*  =  epidermal growth factor receptor, ECOG PS  =  Eastern Cooperative Oncology Group performance status, HR  =  hazard ratio, CI  =  confidence interval.

*Positive of EGFR IHC was according to our scoring method with the best AUC.

## Discussion

This is the first study to demonstrate the influence of total EGFR expression on the IHC interpretation of mutation-specific antibodies, especially L858R, and compare different IHC scoring methods of mutation-specific antibodies by statistical analysis. The scoring method based on the logistic regression model provided the best diagnostic power, and false positive and false negative rates were both decreased in comparison with the scoring system of IHC intensity only. In addition, the positive IHC staining according to the best cut-off point was correlated to a better response rate to EGFR TKIs and a longer PFS. The IHC test of the mutation-specific antibodies is useful for personalized therapy.

The majority of published papers have adopted the IHC intensity scoring method to interpret mutation-specific antibodies, with the definition of a positive IHC result being more than 10% tumor cells with an IHC intensity score of 1+ or more [8], [9], [10], [11], [12]. However, Kato et al. 's study adopted the University of Colorado's IHC H-score criteria, and Kozu et al. adopted another scoring system [13], [14]. The sensitivity and specificity of the mutation-specific antibodies from these scoring systems showed contrasting results, especially in Kitamura et al. 's study where the sensitivity was only 36% for L858R and 40% for E746-A750 deletion [11]. Compared with the above scoring method of IHC intensity (1+), the scoring method of the present study which had the best AUC showed that the false-positive rate decreased from 42% to 23% for L858R, and from 9.8% to 5.4% for E746-A750 deletion, and provided better diagnostic power.

The present study demonstrated that total EGFR expression may affect the IHC interpretation of the L858R antibody. If lung cancer of wild-type *EGFR* had a high total EGFR expression Q score, the L858R mutation antibody had a low level of non-specific stains (Case 4 of Figure 1). Therefore, when using the EGFR mutant-specific antibodies for detecting EGFR mutant lung cancers, side by side IHC with total EGFR antibody is also necessary for the interpretation of the IHC result of mutation-specific antibody. These findings provided an usage of IHC with EGFR total and mutation-specific antibodies to choose the suitable patients for EGFR TKIs treatment.

The use of IHC staining to predict responses to EGFR TKIs has been controversial in previous reports [11], [13], [14]. Kozu Y, et al showed that the sensitivity and specificity of IHC-based *EGFR* mutations to predict response to EGFR TKIs were 63% and 70%, respectively. Besides, the IHC-based mutational status was not significantly correlated to clinical response to EGFR TKI by multivariate analysis [14]. Kato et al. showed that positive IHC staining of the two mutation-specific antibodies produced a nonsignificant trend toward a favorable clinical outcome, including overall survival and PFS [13]. In the present study, the analysis of clinical treatment outcomes confirms the clinical practicability and value of IHC staining according to the cut-off point. Large prospective trials are necessary to prove the clinical value of mutation-specific antibodies.

According to our prior report, patients with complex *EGFR* mutations with the classical mutation pattern had the same response rate, PFS, and overall survival time as those with a single classical mutation [21]. It is important to pick up the tumors harboring complex mutations with classical mutation patterns when considering the treatment with EGFR TKIs. In the present study, the mutation-specific antibodies had a high diagnostic sensitivity (5 of 6, 83.3%) for the tumors harboring complex *EGFR* mutations with classical mutation patterns. IHC with *EGFR* mutant-specific antibodies could therefore be used to screen this type of candidate for the use of EGFR TKIs. This probably implies that the second mutation does not affect the conformation of classical mutation, and therefore EGFR TKI can inhibit the activation of complex-mutant EGFR and the mutant-specific antibody can stain the complex-mutant EGFR.

In the present study, one of four tumors harboring the L747-T751 deletion was also positive to the E746-A750 deletion mutation-specific antibody. Although Kitamura et al. mentioned that this phenomenon may result from the similar conformational composition to the E746-A750 deletion [11], it cannot completely explain why the other three tumors harboring the same *EGFR* mutation, L747-T751 deletion, could not be detected by IHC staining. Future studies are required to elucidate the definite mechanism.

In addition to Del-19 and L858R, EGFR TKIs also lead favorable response in patients with G719 and L861 [23], [24]. It is important for clinical physician to select patients with sensitive mutation to EGFR TKIs. However, the present mutation-specific antibodies were only designed to detect L858R and delE746-A750. The sensitivity and specificity of the IHC for EGFR mutation also did not reach perfect to detect all sensitive mutations. For clinical practice, molecular testing, for example: DNA sequencing, for confirmation may be still necessary if IHC shows negative result.

Five tumors (11.6%, 5 of 43) harbored L858R and two tumors (6.5%, 2 of 31) harbored the E746-A750 deletion could not be detected by IHC staining in the present study, a phenomenon which has also been seen in previous studies [9], [11]. One possible reason may be the heterogeneous component of the cancers may have had an effect, so a random tumor section may have included wild-type *EGFR* cancer cells thereby missing the positive *EGFR* mutation component. In addition, the long-term storage of the paraffin-embedded specimens, as the biological nature may have changed resulting in poor IHC staining. However, in this study we had use pan-cytokeratin stains to confirm the quality of the studied specimens.

In conclusion, total EGFR expression should be included into the interpretation of IHC stain of EGFR L858R antibody. IHC staining of mutation-specific antibodies, which could be routinely practiced in pathology laboratories, is useful to predict EGFR TKI treatment outcome.

## Supporting Information

Table S1 **The genotype and immunohistochemistry results of EGFR mutations.**
(DOCX)

Table S2 **Clinical practice index for the** ***EGFR*** **mutation-specific antibodies of L858R (the corresponding table of predictive probability is listed as Table S4).**
(DOCX)

Table S3 **Clinical practice index for the** ***EGFR*** **mutation-specific antibodies of E746-A750 deletion (the corresponding table of predictive probability is listed as Table S5).**
(DOCX)

Table S4 **The predictive probability of the corresponding table for EGFR mutation-specific antibodies of L858R (cut-off point  = 0.181).**
(DOCX)

Table S5 **The predictive probability of the corresponding table for EGFR mutation-specific antibodies of delE748-A750 (cut-off point  = 0.061).**
(DOCX)

Table S6 **The clinical characteristics and treatment outcomes in the EGFR TKI-treated patients.**
(DOCX)
