# Supplementary material for: Including Total EGFR Staining in Scoring Improves EGFR Mutations Detection by Mutation-Specific Antibodies and EGFR TKIs Response Prediction
Source: PLoS One. 2011 Aug 9;6(8):e23303. doi: 10.1371/journal.pone.0023303 (PMC3153495; doi:10.1371/journal.pone.0023303)
Supplement: Table S1 — The genotype and immunohistochemistry results of EGFR mutations. (DOCX) [file pone.0023303.s001.docx]

**Table S1** The genotype and immunohistochemistry results of *EGFR* mutations

|  |  | **Immunohistochemistry** | | |
| --- | --- | --- | --- | --- |
| **Genotype** |  | **No.** | **Anti-L858R**  **Ab** | **anti-E746-A750**  **Ab** |
| Exon 18 |  |  |  |  |
|  | delE709-T710 insD | 1 | — | — |
|  | E709K + G719A | 1 | — | — |
|  | E709K + G719S | 1 | ＋ | — |
|  | G719A + L861Q | 1 | — | — |
| Exon 19 |  |  |  |  |
|  | delE746-A750 | 20 | — | ＋ |
|  | delE746-A750 | 9 | ＋ | ＋ |
|  | delE746-A750 | 2 | — | — |
|  | delL746-T751insQ | 1 | — | — |
|  | delL747-A750 insP | 1 | ＋ | — |
|  | delL747-T751 | 1 | — | ＋ |
|  | delL747-T751 | 1 | ＋ | — |
|  | delL747-T751 | 2 | — | — |
|  | delL747-P753 | 2 | — | — |
|  | delL747-L754insGC | 1 | — | — |
|  | delT751-I759 insN | 1 | — | — |
| Exon 20 |  |  | — | — |
|  | N771-H773 dupNPH | 1 | — | — |
| Exon 21 |  |  | — | — |
|  | L858R | 32 | ＋ | — |
|  | L858R | 1 | ＋ | ＋ |
|  | L858R | 4 | — | — |
|  | L858R + E709V | 1 | ＋ | — |
|  | L858R + K757N | 1 | ＋ | — |
|  | L858R + R776H | 1 | ＋ | — |
|  | L858R + T790M | 1 | ＋ | ＋ |
|  | L858R + V834L | 1 | ＋ | — |
|  | L858R + V834L | 1 | — | — |
|  | L861Q | 1 | — | — |
|  | L861Q | 1 | ＋ | — |
|  | L861Q + K860I | 1 | — | — |
|  | L861R + R831C | 1 | ＋ | — |
| Wild |  |  |  |  |
|  | wild | 38 | — | — |
|  | wild | 3 | — | ＋ |
|  | wild | 9 | ＋ | — |
