# Supplementary material for: Including Total EGFR Staining in Scoring Improves EGFR Mutations Detection by Mutation-Specific Antibodies and EGFR TKIs Response Prediction
Source: PLoS One. 2011 Aug 9;6(8):e23303. doi: 10.1371/journal.pone.0023303 (PMC3153495; doi:10.1371/journal.pone.0023303)
Supplement: Table S2 — Clinical practice index for the EGFR mutation-specific antibodies of L858R (the corresponding table of predictive probability is listed as Table S4). (DOCX) [file pone.0023303.s002.docx]

**Table S2** Clinical practice index for the *EGFR* mutation-specific antibodies of L858R (the corresponding table of predictive probability is listed as Supplemental Table 4)

| **Total EGFR**  **Q score**  **L858R Q score** | **0** | **5** | **10** | **15** | **20** | **30** | **40** | **50** | **60** | **70** | **80** | **90** | **100** | **120** | **140** | **160** | **180** | **220** | **260** | **300** |
| --- | --- | --- | --- | --- | --- | --- | --- | --- | --- | --- | --- | --- | --- | --- | --- | --- | --- | --- | --- | --- |
| **0** | **-** | **-** | **-** | **-** | **-** | **-** | **-** | **-** | **-** | **-** | **-** | **-** | **-** | **-** | **-** | **-** | **-** | **-** | **-** | **-** |
| **5** | **-** | **-** | **-** | **-** | **-** | **-** | **-** | **-** | **-** | **-** | **-** | **-** | **-** | **-** | **-** | **-** | **-** | **-** | **-** | **-** |
| **10** | **-** | **-** | **-** | **-** | **-** | **-** | **-** | **-** | **-** | **-** | **-** | **-** | **-** | **-** | **-** | **-** | **-** | **-** | **-** | **-** |
| **15** | **＋** | **＋** | **＋** | **-** | **-** | **-** | **-** | **-** | **-** | **-** | **-** | **-** | **-** | **-** | **-** | **-** | **-** | **-** | **-** | **-** |
| **20** | **＋** | **＋** | **＋** | **＋** | **＋** | **-** | **-** | **-** | **-** | **-** | **-** | **-** | **-** | **-** | **-** | **-** | **-** | **-** | **-** | **-** |
| **30** | **＋** | **＋** | **＋** | **＋** | **＋** | **＋** | **＋** | **-** | **-** | **-** | **-** | **-** | **-** | **-** | **-** | **-** | **-** | **-** | **-** | **-** |
| **40** | **＋** | **＋** | **＋** | **＋** | **＋** | **＋** | **＋** | **＋** | **＋** | **＋** | **-** | **-** | **-** | **-** | **-** | **-** | **-** | **-** | **-** | **-** |
| **50** | **＋** | **＋** | **＋** | **＋** | **＋** | **＋** | **＋** | **＋** | **＋** | **＋** | **＋** | **＋** | **＋** | **-** | **-** | **-** | **-** | **-** | **-** | **-** |
| **60** | **＋** | **＋** | **＋** | **＋** | **＋** | **＋** | **＋** | **＋** | **＋** | **＋** | **＋** | **＋** | **＋** | **＋** | **-** | **-** | **-** | **-** | **-** | **-** |
| **70** | **＋** | **＋** | **＋** | **＋** | **＋** | **＋** | **＋** | **＋** | **＋** | **＋** | **＋** | **＋** | **＋** | **＋** | **＋** | **-** | **-** | **-** | **-** | **-** |
| **80** | **＋** | **＋** | **＋** | **＋** | **＋** | **＋** | **＋** | **＋** | **＋** | **＋** | **＋** | **＋** | **＋** | **＋** | **＋** | **＋** | **-** | **-** | **-** | **-** |
| **81** | **＋** | **＋** | **＋** | **＋** | **＋** | **＋** | **＋** | **＋** | **＋** | **＋** | **＋** | **＋** | **＋** | **＋** | **＋** | **＋** | **＋** | **-** | **-** | **-** |
| **100** | **＋** | **＋** | **＋** | **＋** | **＋** | **＋** | **＋** | **＋** | **＋** | **＋** | **＋** | **＋** | **＋** | **＋** | **＋** | **＋** | **＋** | **＋** | **-** | **-** |
| **120** | **＋** | **＋** | **＋** | **＋** | **＋** | **＋** | **＋** | **＋** | **＋** | **＋** | **＋** | **＋** | **＋** | **＋** | **＋** | **＋** | **＋** | **＋** | **＋** | **-** |
| **140** | **＋** | **＋** | **＋** | **＋** | **＋** | **＋** | **＋** | **＋** | **＋** | **＋** | **＋** | **＋** | **＋** | **＋** | **＋** | **＋** | **＋** | **＋** | **＋** | **＋** |
| **180** | **＋** | **＋** | **＋** | **＋** | **＋** | **＋** | **＋** | **＋** | **＋** | **＋** | **＋** | **＋** | **＋** | **＋** | **＋** | **＋** | **＋** | **＋** | **＋** | **＋** |
| **220** | **＋** | **＋** | **＋** | **＋** | **＋** | **＋** | **＋** | **＋** | **＋** | **＋** | **＋** | **＋** | **＋** | **＋** | **＋** | **＋** | **＋** | **＋** | **＋** | **＋** |
| **260** | **＋** | **＋** | **＋** | **＋** | **＋** | **＋** | **＋** | **＋** | **＋** | **＋** | **＋** | **＋** | **＋** | **＋** | **＋** | **＋** | **＋** | **＋** | **＋** | **＋** |
| **300** | **＋** | **＋** | **＋** | **＋** | **＋** | **＋** | **＋** | **＋** | **＋** | **＋** | **＋** | **＋** | **＋** | **＋** | **＋** | **＋** | **＋** | **＋** | **＋** | **＋** |
