# Supplementary material for: Including Total EGFR Staining in Scoring Improves EGFR Mutations Detection by Mutation-Specific Antibodies and EGFR TKIs Response Prediction
Source: PLoS One. 2011 Aug 9;6(8):e23303. doi: 10.1371/journal.pone.0023303 (PMC3153495; doi:10.1371/journal.pone.0023303)
Supplement: Table S3 — Clinical practice index for the EGFR mutation-specific antibodies of E746-A750 deletion (the corresponding table of predictive probability is listed as Table S5). (DOCX) [file pone.0023303.s003.docx]

**Table S3** Clinical practice index for the *EGFR* mutation-specific antibodies of E746-A750 deletion (the corresponding table of predictive probability is listed as Supplemental Table 5)

| **Total EGFR expression**  **intensity**  **del E746-A750 Q score** | **0** | **1+**  **(Weak)** | **2+**  **(Moderate)** | **3+**  **(Strong)** |
| --- | --- | --- | --- | --- |
| **0** | **-** | **-** | **-** | **-** |
| **5** | **-** | **-** | **-** | **-** |
| **10** | **-** | **-** | **-** | **-** |
| **15** | **＋** | **＋** | **＋** | **＋** |
| **20** | **＋** | **＋** | **＋** | **＋** |
| **30** | **＋** | **＋** | **＋** | **＋** |
| **40** | **＋** | **＋** | **＋** | **＋** |
| **50** | **＋** | **＋** | **＋** | **＋** |
| **60** | **＋** | **＋** | **＋** | **＋** |
| **70** | **＋** | **＋** | **＋** | **＋** |
| **80** | **＋** | **＋** | **＋** | **＋** |
| **81** | **＋** | **＋** | **＋** | **＋** |
| **100** | **＋** | **＋** | **＋** | **＋** |
| **120** | **＋** | **＋** | **＋** | **＋** |
| **140** | **＋** | **＋** | **＋** | **＋** |
| **180** | **＋** | **＋** | **＋** | **＋** |
| **220** | **＋** | **＋** | **＋** | **＋** |
| **260** | **＋** | **＋** | **＋** | **＋** |
| **300** | **＋** | **＋** | **＋** | **＋** |
