# Supplementary material for: Including Total EGFR Staining in Scoring Improves EGFR Mutations Detection by Mutation-Specific Antibodies and EGFR TKIs Response Prediction
Source: PLoS One. 2011 Aug 9;6(8):e23303. doi: 10.1371/journal.pone.0023303 (PMC3153495; doi:10.1371/journal.pone.0023303)
Supplement: Table S4 — The predictive probability of the corresponding table for EGFR mutation-specific antibodies of L858R (cut-off point = 0.181). (DOCX) [file pone.0023303.s004.docx]

**Table S4** The predictive probability of the corresponding table for *EGFR* mutation-specific antibodies of L858R (cut-off point = 0.181)

**(A)**

| **Total EGFR**  **Q score**  **L858R Q score** | **0** | **5** | **10** | **15** | **20** | **30** | **40** | **50** | **60** | **70** | **80** | **90** | **100** | **120** | **140** | **160** | **180** | **220** | **260** | **300** |
| --- | --- | --- | --- | --- | --- | --- | --- | --- | --- | --- | --- | --- | --- | --- | --- | --- | --- | --- | --- | --- |
| **0** | 0.120 | 0.115 | 0.110 | 0.105 | 0.101 | 0.092 | 0.084 | 0.077 | 0.070 | 0.064 | 0.058 | 0.053 | 0.048 | 0.040 | 0.033 | 0.027 | 0.022 | 0.015 | 0.010 | 0.007 |
| **5** | 0.135 | 0.129 | 0.124 | 0.118 | 0.113 | 0.104 | 0.095 | 0.086 | 0.079 | 0.072 | 0.065 | 0.060 | 0.054 | 0.045 | 0.037 | 0.031 | 0.025 | 0.017 | 0.011 | 0.008 |
| **10** | 0.151 | 0.145 | 0.138 | 0.133 | 0.127 | 0.116 | 0.106 | 0.097 | 0.089 | 0.081 | 0.074 | 0.067 | 0.061 | 0.051 | 0.042 | 0.035 | 0.029 | 0.019 | 0.013 | 0.009 |
| **15** | **0.168** | **0.161** | **0.155** | 0.148 | 0.142 | 0.130 | 0.119 | 0.109 | 0.100 | 0.091 | 0.083 | 0.076 | 0.069 | 0.057 | 0.048 | 0.039 | 0.032 | 0.022 | 0.015 | 0.010 |
| **20** | **0.187** | **0.180** | **0.173** | **0.165** | **0.159** | 0.146 | 0.134 | 0.123 | 0.112 | 0.103 | 0.094 | 0.086 | 0.078 | 0.065 | 0.054 | 0.044 | 0.037 | 0.025 | 0.017 | 0.011 |
| **30** | **0.230** | **0.221** | **0.213** | **0.205** | **0.197** | **0.181** | **0.167** | 0.153 | 0.141 | 0.129 | 0.118 | 0.108 | 0.099 | 0.083 | 0.069 | 0.057 | 0.047 | 0.032 | 0.022 | 0.015 |
| **40** | **0.279** | **0.269** | **0.260** | **0.250** | **0.241** | **0.223** | **0.206** | **0.190** | **0.175** | **0.161** | 0.148 | 0.136 | 0.125 | 0.105 | 0.087 | 0.073 | 0.060 | 0.041 | 0.028 | 0.019 |
| **50** | **0.334** | **0.323** | **0.313** | **0.302** | **0.292** | **0.271** | **0.252** | **0.234** | **0.216** | **0.200** | **0.184** | **0.170** | **0.156** | 0.131 | 0.110 | 0.092 | 0.077 | 0.053 | 0.036 | 0.024 |
| **60** | **0.395** | **0.383** | **0.371** | **0.359** | **0.348** | **0.326** | **0.304** | **0.283** | **0.263** | **0.245** | **0.227** | **0.209** | **0.193** | **0.164** | 0.138 | 0.116 | 0.097 | 0.067 | 0.046 | 0.031 |
| **70** | **0.458** | **0.446** | **0.433** | **0.421** | **0.409** | **0.385** | **0.362** | **0.339** | **0.317** | **0.296** | **0.275** | **0.256** | **0.237** | **0.203** | **0.173** | 0.146 | 0.123 | 0.086 | 0.059 | 0.040 |
| **80** | **0.523** | **0.510** | **0.498** | **0.486** | **0.473** | **0.448** | **0.424** | **0.399** | **0.376** | **0.353** | **0.330** | **0.308** | **0.287** | **0.248** | **0.213** | **0.181** | 0.153 | 0.108 | 0.075 | 0.052 |
| **81** | **0.587** | **0.575** | **0.563** | **0.550** | **0.538** | **0.513** | **0.488** | **0.463** | **0.438** | **0.414** | **0.390** | **0.366** | **0.343** | **0.300** | **0.260** | **0.223** | **0.190** | 0.136 | 0.096 | 0.066 |
| **100** | **0.648** | **0.637** | **0.625** | **0.613** | **0.602** | **0.577** | **0.553** | **0.528** | **0.503** | **0.478** | **0.453** | **0.428** | **0.404** | **0.357** | **0.313** | **0.271** | **0.234** | **0.170** | 0.120 | 0.084 |
| **120** | **0.756** | **0.747** | **0.737** | **0.728** | **0.717** | **0.697** | **0.675** | **0.653** | **0.630** | **0.606** | **0.582** | **0.558** | **0.533** | **0.483** | **0.433** | **0.385** | **0.339** | **0.256** | **0.187** | 0.134 |
| **140** | **0.839** | **0.832** | **0.825** | **0.818** | **0.810** | **0.794** | **0.778** | **0.760** | **0.741** | **0.722** | **0.701** | **0.680** | **0.657** | **0.611** | **0.563** | **0.513** | **0.463** | **0.366** | **0.279** | **0.206** |
| **180** | **0.937** | **0.934** | **0.930** | **0.927** | **0.924** | **0.916** | **0.908** | **0.900** | **0.890** | **0.880** | **0.869** | **0.857** | **0.844** | **0.816** | **0.784** | **0.749** | **0.709** | **0.621** | **0.523** | **0.424** |
| **220** | **0.977** | **0.975** | **0.974** | **0.973** | **0.972** | **0.969** | **0.966** | **0.962** | **0.958** | **0.954** | **0.949** | **0.944** | **0.939** | **0.926** | **0.911** | **0.894** | **0.873** | **0.822** | **0.756** | **0.675** |
| **260** | **0.992** | **0.991** | **0.991** | **0.990** | **0.990** | **0.989** | **0.988** | **0.986** | **0.985** | **0.983** | **0.982** | **0.980** | **0.978** | **0.973** | **0.967** | **0.960** | **0.951** | **0.929** | **0.898** | **0.855** |
| **300** | **0.997** | **0.997** | **0.997** | **0.997** | **0.996** | **0.996** | **0.996** | **0.995** | **0.995** | **0.994** | **0.993** | **0.993** | **0.992** | **0.990** | **0.988** | **0.985** | **0.982** | **0.974** | **0.961** | **0.943** |
