# Supplementary material for: Including Total EGFR Staining in Scoring Improves EGFR Mutations Detection by Mutation-Specific Antibodies and EGFR TKIs Response Prediction
Source: PLoS One. 2011 Aug 9;6(8):e23303. doi: 10.1371/journal.pone.0023303 (PMC3153495; doi:10.1371/journal.pone.0023303)
Supplement: Table S5 — The predictive probability of the corresponding table for EGFR mutation-specific antibodies of delE748-A750 (cut-off point = 0.061). (DOCX) [file pone.0023303.s005.docx]

**Table S5** The predictive probability of the corresponding table for *EGFR* mutation-specific antibodies of delE748-A750 (cut-off point = 0.061)

| **Total EGFR expression**  **intensity**  **del E746-A750 Q score** | **0** | **1+**  **(Weak)** | **2+**  **(Moderate)** | **3+**  **(Strong)** |
| --- | --- | --- | --- | --- |
| **0** | 0.043 | 0.044 | 0.046 | 0.048 |
| **5** | 0.057 | 0.059 | 0.061 | 0.063 |
| **10** | **0.075** | **0.077** | **0.080** | **0.083** |
| **15** | **0.098** | **0.101** | **0.105** | **0.108** |
| **20** | **0.127** | **0.131** | **0.136** | **0.140** |
| **30** | **0.321** | **0.330** | **0.338** | **0.347** |
| **40** | **0.606** | **0.615** | **0.624** | **0.633** |
| **50** | **0.834** | **0.839** | **0.844** | **0.849** |
| **60** | **0.942** | **0.944** | **0.946** | **0.948** |
| **70** | **0.982** | **0.982** | **0.983** | **0.983** |
| **80** | **0.994** | **0.994** | **0.995** | **0.995** |
| **81** | **0.998** | **0.998** | **0.998** | **0.998** |
| **100** | **0.999** | **0.999** | **0.999** | **1.000** |
| **120** | **1.000** | **1.000** | **1.000** | **1.000** |
| **140** | **1.000** | **1.000** | **1.000** | **1.000** |
| **180** | **1.000** | **1.000** | **1.000** | **1.000** |
| **220** | **1.000** | **1.000** | **1.000** | **1.000** |
| **260** | **1.000** | **1.000** | **1.000** | **1.000** |
| **300** | **1.000** | **1.000** | **1.000** | **1.000** |
