# Supplementary material for: Including Total EGFR Staining in Scoring Improves EGFR Mutations Detection by Mutation-Specific Antibodies and EGFR TKIs Response Prediction
Source: PLoS One. 2011 Aug 9;6(8):e23303. doi: 10.1371/journal.pone.0023303 (PMC3153495; doi:10.1371/journal.pone.0023303)
Supplement: Table S6 — The clinical characteristics and treatment outcomes in the EGFR TKI-treated patients. (DOCX) [file pone.0023303.s006.docx]

**Table S6** The clinical characteristics and treatment outcomes in the EGFR TKI-treated patients

| No. | Sex | Age | Smoking | *EGFR* mutation | Anti-L858R  Ab | Anti-E746-A750  Ab | EGFR TKI | EGFR TKI  response | PFS | Progression |
| --- | --- | --- | --- | --- | --- | --- | --- | --- | --- | --- |
| 1 | M | 63.4 | Yes | delE746-A750 | — | ＋ | erlotinib | PR | 20.5 | progression |
| 2 | M | 60.6 | Yes | delE746-A750 | — | ＋ | gefitinib | PR | 26.9 | progression |
| 3 | F | 56.3 | No | delE746-A750 | ＋ | ＋ | gefitinib | PR | 9.0 | progression |
| 4 | F | 53.9 | No | delE746-A750 | — | ＋ | gefitinib | PR | 11.6 | progression |
| 5 | F | 63.3 | No | delE746-A750 | — | ＋ | gefitinib | PR | 9.0 | progression |
| 6 | M | 52.0 | Yes | delE746-A750 | — | ＋ | erlotinib | PR | 35.9 | No |
| 7 | M | 64.7 | No | delE746-A750 | — | ＋ | gefitinib | PR | 10.7 | progression |
| 8 | F | 71.1 | No | delE746-A750 | — | ＋ | gefitinib | PR | 17.1 | No |
| 9 | M | 61.2 | No | delE746-A750 | — | ＋ | erlotinib | PR | 13.8 | No |
| 10 | M | 76.7 | Yes | delE746-A750 | — | ＋ | gefitinib | PD | 3.3 | progression |
| 11 | M | 66.9 | Yes | del L747-P753 | — | — | gefitinib | PR | 4.7 | progression |
| 12 | F | 71.5 | No | del L747-T751 insQ | — | — | gefitinib | PR | 14.7 | progression |
| 13 | F | 75.7 | No | L858R | ＋ | — | gefitinib | PR | 6.1 | progression |
| 14 | F | 62.4 | No | L858R | ＋ | — | gefitinib | PR | 5.2 | progression |
| 15 | F | 70.9 | No | L858R | ＋ | — | gefitinib | PR | 45.2 | progression |
| 16 | M | 68.3 | Yes | L858R | — | — | gefitinib | SD | 46.4 | progression |
| 17 | F | 72.4 | No | L858R | ＋ | — | erlotinib | PR | 12.0 | progression |
| 18 | F | 74.1 | No | L858R | ＋ | — | gefitinib | PR | 7.2 | progression |
| 19 | F | 70.0 | No | L858R | ＋ | — | gefitinib | SD | 1.8 | No |
| 20 | F | 65.6 | No | L858R | ＋ | — | erlotinib | PR | 28.3 | No |
| 21 | M | 76.9 | No | L858R | ＋ | — | erlotinib | PR | 20.7 | No |
| 22 | F | 67.1 | No | delE709-T710 insD | — | — | gefitinib | PR | 5.1 | progression |
| 23 | F | 76.1 | No | R831C + L861R | ＋ | — | gefitinib | PR | 15.9 | No |
| 24 | F | 60.8 | No | wild | — | — | gefitinib | PD | 4.7 | progression |
| 25 | M | 67.3 | Yes | wild | — | — | gefitinib | PD | 3.0 | progression |
| 26 | M | 58.2 | Yes | wild | ＋ | — | gefitinib | PD | 1.7 | progression |
| 27 | M | 78.0 | Yes | wild | ＋ | — | gefitinib | PD | 0.5 | progression |
| 28 | F | 41.4 | No | wild | — | — | gefitinib | PD | 2.3 | progression |
| 29 | M | 66.1 | Yes | wild | ＋ | — | erlotinib | SD | 5.3 | No |
| 30 | F | 54.2 | No | wild | — | — | gefitinib | PD | 1.5 | progression |
| 31 | F | 44.6 | No | wild | — | — | gefitinib | PD | 1.3 | progression |
| 32 | F | 27.2 | No | wild | — | — | gefitinib | PR | 11.4 | progression |
| 33 | M | 66.6 | Yes | wild | — | — | erlotinib | PD | 1.7 | progression |
| 34 | M | 65.9 | Yes | wild | — | — | erlotinib | PR | 19.3 | progression |
| 35 | M | 70.2 | Yes | wild | — | — | erlotinib | PD | 0.9 | progression |
| 36 | M | 47.6 | No | wild | — | — | erlotinib | PD | 0.7 | progression |
| 37 | M | 64.0 | Yes | wild | — | — | erlotinib | SD | 5.1 | progression |

PFS: progression-free survival, PR:partital respsponse, SD: stable disease, PD: progressive disease
